# Pyrazolo[1,5-*a*]pyrimidine as a Prominent Framework for Tropomyosin Receptor Kinase (Trk) Inhibitors—Synthetic Strategies and SAR Insights

**DOI:** 10.3390/molecules29153560

**Published:** 2024-07-29

**Authors:** Amol T. Mahajan, Shivani, Ashok Kumar Datusalia, Carmine Coluccini, Paolo Coghi, Sandeep Chaudhary

**Affiliations:** 1 Laboratory of Bioactive Heterocycles and Catalysis (BHC Lab), Department of Medicinal Chemistry, National Institute of Pharmaceutical Education and Research-Raebareli (Transit Campus), Bijnor–Sisendi Road, Near CRPF Base Camp, Sarojini Nagar, Lucknow 226002, India; amolmahajan181@gmail.com (A.T.M.); shivani.ss2606@gmail.com (S.); 2 Laboratory of Molecular Neurotherapeutics, Department of Pharmacology and Toxicology, National Institute of Pharmaceutical Education and Research-Raebareli (Transit Campus), Bijnor–Sisendi Road, Near CRPF Base Camp, Sarojini Nagar, Lucknow 226002, India; ashok.datusalia@niperraebareli.edu.in; 3 Institute of New Drug Development, College of Medicine, China Medical University, No. 91, Hsueh-Shih Road, Taichung 40402, Taiwan; 4 Laboratory for Drug Discovery from Natural Resources & Industrialization, School of Pharmacy, Macau University of Science and Technology, Avenida Wai Long, Taipa, Macau 999078, China

**Keywords:** TRK, cancer, anticancer, synthetic strategies, SAR

## Abstract

Tropomyosin receptor kinases (Trks) are transmembrane receptor tyrosine kinases named TrkA, TrkB, and TrkC and encoded by the NTRK1, NTRK2, and NTRK3 genes, respectively. These kinases have attracted significant attention and represent a promising therapeutic target for solid tumor treatment due to their vital role in cellular signaling pathways. First-generation TRK inhibitors, i.e., Larotrectinib sulfate and Entrectinib, received clinical approval in 2018 and 2019, respectively. However, the use of these inhibitors was significantly limited because of the development of resistance due to mutations. Fortunately, the second-generation Trk inhibitor Repotrectinib (TPX-0005) was approved by the FDA in November 2023, while Selitrectinib (Loxo-195) has provided an effective solution to this issue. Another macrocycle-based analog, along with many other TRK inhibitors, is currently in clinical trials. Two of the three marketed drugs for NTRK fusion cancers feature a pyrazolo[1,5-*a*] pyrimidine nucleus, prompting medicinal chemists to develop numerous novel pyrazolopyrimidine-based molecules to enhance clinical applications. This article focuses on a comprehensive review of chronological synthetic developments and the structure–activity relationships (SAR) of pyrazolo[1,5-*a*]pyrimidine derivatives as Trk inhibitors. This article will also provide comprehensive knowledge and future directions to the researchers working in the field of medicinal chemistry by facilitating the structural modification of pyrazolo [1,5-*a*]pyrimidine derivatives to synthesize more effective novel chemotherapeutics as TRK inhibitors.

## 1. Introduction

**Figure 1:**
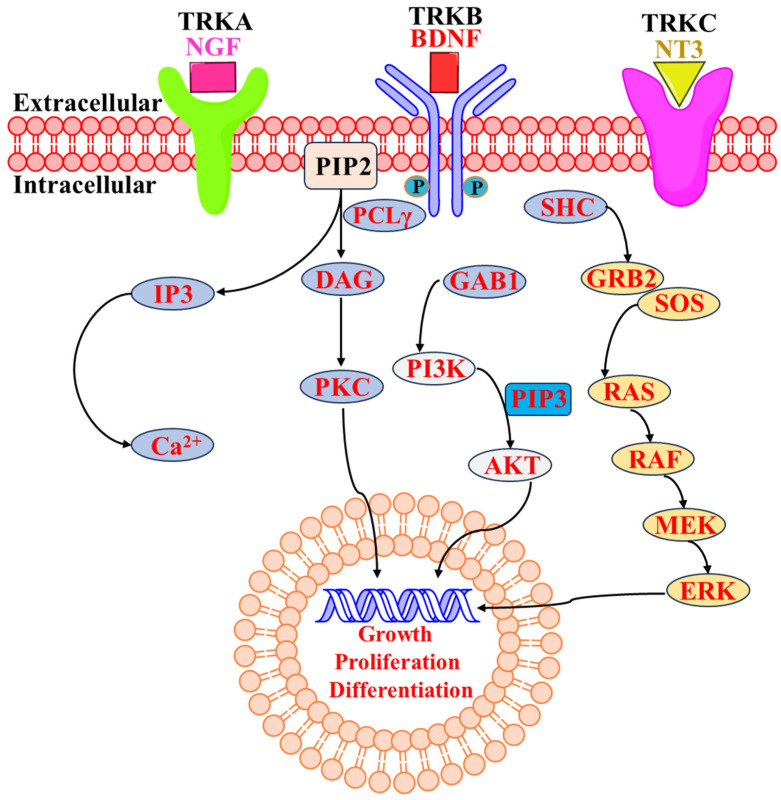
Schematic presentation of Trk receptors and their major signal transduction pathway [53,54].

The tropomyosin receptor kinases (Trks) are part of the cell surface receptor tyrosine kinase (RTK) family. Tropomyosin receptor kinases A, B, and C (TrkA, TrkB, and TrkC) are encoded by the genes encoding neurotrophic receptor tyrosine kinase 1, 2, and 3 (NTRK1, NTRK2, and NTRK3). NTRK1, NTRK2, and NTRK3 are homologous genes that encode receptor tyrosine kinases involved in neural development and function. In the mammalian nervous system, these Trks are crucial for synaptic plasticity and neuronal growth [1]. Transmembrane receptor proteins, or TRKs, are composed of three domains: an intracellular domain with activity, a transmembrane domain, and an extracellular ligand-binding domain [2]. The overall sequence homology of the kinase domains of TrkA, TrkB, and TrkC ranges from 71.9% to 78.3%, with TrkB and TrkC exhibiting the highest degree of similarity [3,4]. Many ligands that activate the receptors distinguish the three subtypes of TRKs (Figure 1) from one another. Mainly, nerve growth factor (NGF), neurotrophin-7, and neurotrophin-6 bind to TrkA, and activation of TrkA triggers various signaling pathways involved in cell survival, differentiation, and growth, particularly in neurons and neuroblastomas [5]. On the other hand, brain-derived neurotrophic factor (BDNF) and neurotrophin-4/5 bind to TrkB and atypical activation of TrkB due to mutations or fusions involving NTRK2 can contribute to oncogenic transformation in certain cancers. [6]. Neurotrophin-3 (NT3) is the only nerve growth factor that particularly binds to TrkC and alterations such as mutations or fusions involving NTRK3 can lead to constitutive activation of TrkC, contributing to oncogenesis in specific cancer contexts. [7,8]. When neurotrophins are recognized extracellularly by their cognate Trk receptor, the cytosolic catalytic domain of the receptor’s tyrosine residues becomes dimerized and transphosphorylated. This leads to the activation of multiple downstream signal transduction pathways, such as PI3K-AKT and RAS-MAPK, and regulates the nervous system and neuronal cell cycle, proliferation, apoptosis, survival, and differentiation [9,10,11,12,13]. However, in the pathophysiology of malignant cancer in humans, Trks can be seen to be active through NTRK gene fusions [13,14]. Over 100 distinct NTRK fusion partners have been found in various malignancies to date, with the first NTRK fusion gene, TPM3-NTRK1, found in human colorectal carcinoma in 1982 [15,16]. It has been suggested that TRK proteins have been considered efficient “pan-cancer” targets for the treatment of various cancers harboring NTRK fusions [17,18]. A broad spectrum of cancer types, i.e., NTRK fusions, are found across a wide variety of cancer types [19,20]. NTRK fusions are often driver mutations in the cancers in which they occur. A driver mutation is a genetic alteration that provides a selective growth advantage to cancer cells, contributing to their proliferation and survival [21,22]. Preclinical and clinical studies have demonstrated that cancers with NTRK fusions are highly sensitive to Trk inhibitors. Trk inhibitors specifically target the abnormal TRK fusion proteins, inhibiting their function and thereby inhibiting growth [23]. Trk inhibitors have shown remarkable clinical efficacy in patients with cancers harboring NTRK fusions. Responses have been durable and often lead to significant clinical benefits, including tumor shrinkage and prolonged progression-free survival. This makes Trk inhibitors promising options for patients with advanced or metastatic cancers that harbor these specific genetic alterations [24,25]. Acute myeloid leukemia (AML), lung cancer, cylindrical tumors, breast cancer, human neuroblastoma, colon cancer, melanoma, thyroid cancer, ovarian cancer, prostate cancer, large cell neuroendocrine tumors, pediatrics gliomas and astrocytomas, intrahepatic cholangiocarcinomas, gastrointestinal stromal tumors, gallbladder adenocarcinomas, pancreatic carcinomas, and many other cancers have been associated with oncogenic forms of NTRK [26,27,28,29,30,31,32,33,34,35,36,37,38,39,40,41,42,43,44]. Therefore, using TRK small molecule inhibitors is the primary strategy for targeting NTRK fusion genes. Over the past decade, TRKs have been an area of interest for many researchers/scientists and pharmaceutical industries worldwide. Thus, TRKs have become an important anticancer target for extensive investigation. Eventually, many efforts have been made in the development of anticancer agents, leading to an increase in the large number of TRK inhibitors. NTRK fusion is not only involved in cancer metastasis but also in various other diseases such as asthma [45], interstitial cystitis [46], inflammatory diseases such as ulcerative colitis and Crohn’s disease [47], atopic dermatitis [48], eczema and psoriasis [49], pain [50], Alzheimer’s disease [51], and inflammation [52].

**Figure 2:**
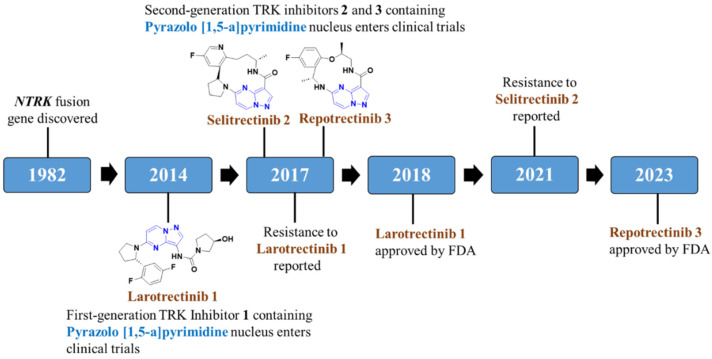
Development of NTRK fusion inhibitors consisting of a pyrazolo[1,5-*a*]pyrimidine moiety.

Larotrectinib (brand name Vitrakvi^®^, 2018, Bayer AG; Leverkusen, Germany) and Entrectinib (brand name Rozlytrek^®^, 2019, Genentech Inc. Group of Roche pharma, Basel, Switzerland) are first-generation Trk inhibitors on the market (Figure 2) [55,56,57]. Both drugs were found to have massive clinical findings against Trk; however, the emergence of drug resistance in clinics restricted their extensive use. In continuation, the emergence of drug resistance further led to the identification of a new generation of drugs to combat these resistances, i.e., second-generation Trk inhibitors were discovered. Repotrectinib (TPX-0005) was approved by the FDA in November 2023 while Selitrectinib (Loxo-195), the macrocycle-based representative, along with many other inhibitors, is currently being used in clinical trials [58,59,60]. Amongst the three marketed drugs for NTRK fusion cancer, two of them consist of a pyrazolo[1,5-*a*]pyrimidine nucleus (PP), which leads to the development of many more small molecules consisting of a PP moiety. Thus, in this review, we have discussed the importance of the PP moiety in the development of novel NTRK fusion inhibitors by incorporating various synthetic strategies and structure–activity relationship (SAR) analysis.

## 2. Synthetic Strategies

**Figure 3:**
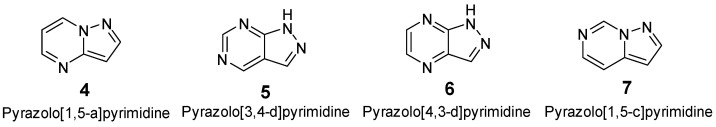
Different fundamental isoforms of pyrazolopyrimidines.

Pyrazolopyrimidine is a fused heterocycle consisting of two N-heterocycles, i.e., pyrazole and pyrimidine. It is a prominent scaffold in medicinal chemistry along with pesticide and therapeutics. It has been engineered to exhibit specific modes of action, including activities as central nervous system (CNS) agents, anti-bacterial agents, anti-inflammatory agents, anti-cancer agents, anti-fungal agents, antiviral agents, and ligands for estrogen receptors [61,62]. It has also been found in multiple kinase inhibitors such as protein kinase, CDK, Trk, EGFR, and FGFR [63]. The basic structures of pyrazolopyrimidine are primarily divided into four categories: pyrazolo[1,5-*a*]pyrimidine, pyrazolo[3,4-*d*]pyrimidine, pyrazolo[4,3-*d*]pyrimidine, and pyrazolo[1,5-*c*]pyrimidine [64] (Figure 3).

**Figure 4:**
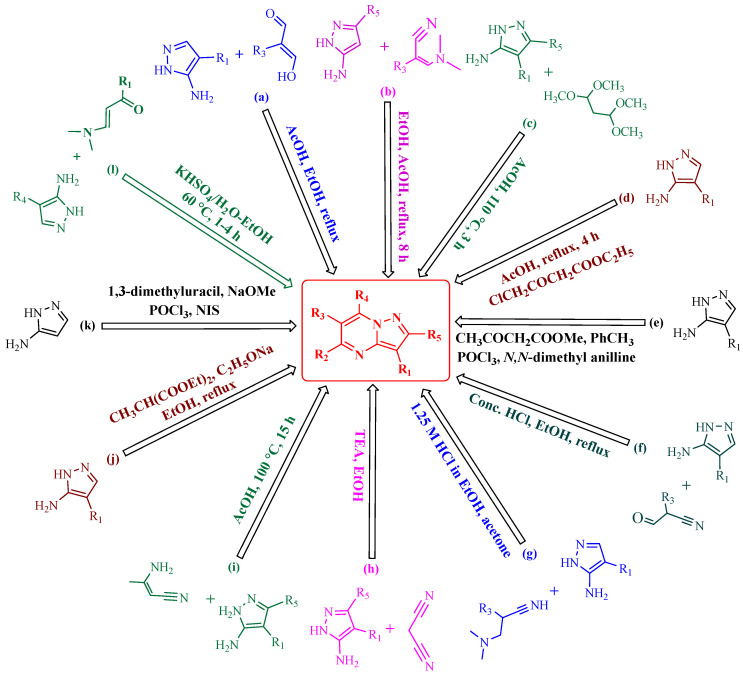
Schematic representation of various synthetic strategies to obtain the PP skeleton.

Numerous methodologies have been reported in the literature for the synthesis of the PP scaffold; the majority of which entail an electrophilic reaction with 1*H*-pyrazole-5-amine and its derivatives. Due to its prevalence in NTRK fusion inhibitors, we have also covered the synthesis and biological action of PP among the four isoforms. PP can be readily synthesized by using enamines, *β*-diketones, and *α*,*β*-unsaturated ketones reacted with 1*H*-pyrazole-5-amine [65]. Herein, we have discussed various synthetic strategies for the synthesis of PP, which have been represented schematically in Figure 4.

Fraley et al. have synthesized the desired novel PP by treating a 4-phenylpyrazolamine derivative with phenyl acrylaldehyde in the presence of acetic acid and ethanol as a solvent (route a) [66]. Elmaati and El-Taweel have used the substituted enaminones and reacted them with 5-aminopyrazole to furnish the PP derivatives (route b) [67].

Campton et al. invented the reaction of 5-aminopyrazole derivatives with 1,1,3,3- tetramethoxypropane in acidic conditions to obtain the product PP (route c) [68]. Li and colleagues synthesized PP by performing the reaction between 5-aminopyrazole and ethyl 4-chloroacetoacetate in the presence of acetone as a solvent under refluxed conditions (route d) [69]. Paruch et al. invented the one-pot synthesis of new PP analogs by reacting 1*H*-pyrazol-5-amine derivatives with methyl-3-oxobutanoate, phosphorus oxychloride, and N,N dimethylaniline under inert conditions (route e) [70]. Frey and co-workers have successfully synthesized novel 7-aminopyrazolo[1,5-*a*]pyrimidines through a cyclocondensation reaction between 5-aminopyrazole derivatives with 3-oxo-2-phenyl propanenitrile (route f) [71]. Gommermann et al. performed the cyclization reaction of 1*H*-pyrazol-5-amine derivatives with 3-(dimethylamino)-2-(4-nitrophenyl) acrylonitrile to obtain the substituted PP under acidic conditions (1.25 M HCl in ethanol) in acetone (route g) [72]. Enany and co-workers acquired the final compound PP by reacting 5-aminopyrazole and malononitrile in the presence of an organic base such as triethylamine and ethanol as a solvent (route h) [73]. Ivachtchenko et al. carried out the reaction of amino-substituted 1*H*-pyrazol-5-amine and 3-aminobut-2-enenitrile in acetic acid to furnish the PP compound (route i) [74]. Kosugi et al. acquired a novel PP derivative by reacting amino pyrazole with a 2-substituted malonic acid diester in the presence of sodium ethoxide in ethanol under reflux conditions (route j) [75]. Dwyer and colleagues carried out a condensation reaction of 5-aminopyrazole with 1,3-dimethyluracil in the presence of phosphorus oxychloride, sodium methoxide, and N-iodosuccinimide to yield the PP derivatives (route k) [76]. Vishwakarma et al. carried out the reaction of 5-aminopyrazole with α,β-unsaturated carbonyl compounds in the presence of KHSO_4_ in an aqueous medium at 60 °C to obtain the desired product PP (route l) [77].

## 3. Pyrazolo[1,5-a]pyrimidine as TRK Inhibitors

**Figure 5:**
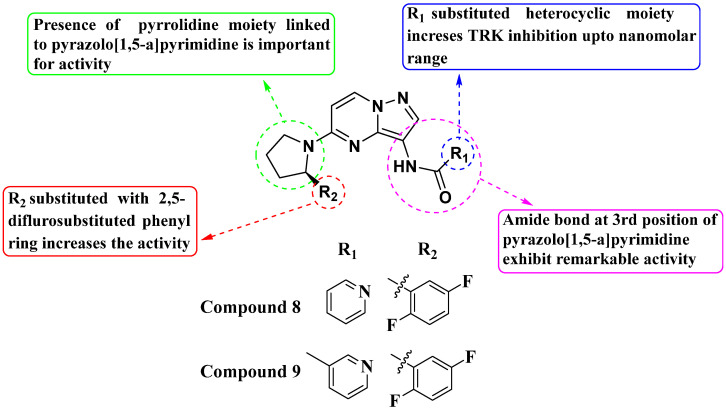
SAR study of pyrrolidine-linked PP derivatives as TrkA inhibitors.

Julia Hass and her colleagues introduced a new series of picolinamide-substituted PP derivatives in 2010 and evaluated them for Trk inhibition (see Figure 5). Among the 105 compounds studied, compounds **8** and **9** demonstrated excellent enzymatic inhibition of TrkA, each with an IC_50_ value of 1.7 nM. The presence of the amide bond of picolinamide at the third position of the pyrazolo[1,5-a]pyrimidine ring significantly enhanced activity. Furthermore, substitution with a 2,5-difluorophenyl-substituted pyrrolidine at the fifth position further increased Trk inhibition activity. Picolinamide can be replaced with various heterocyclic moieties to potentially enhance activity in the nanomolar range. The strategic positioning of the picolinamide moiety and further substitution with a 2,5-difluorophenyl-substituted pyrrolidine highlight promising directions for enhancing Trk inhibition. Future research focusing on optimizing these molecular structures with other heterocycles could possibly lead to even more potent Trk inhibitors with potential therapeutic applications [78].

**Figure 6:**
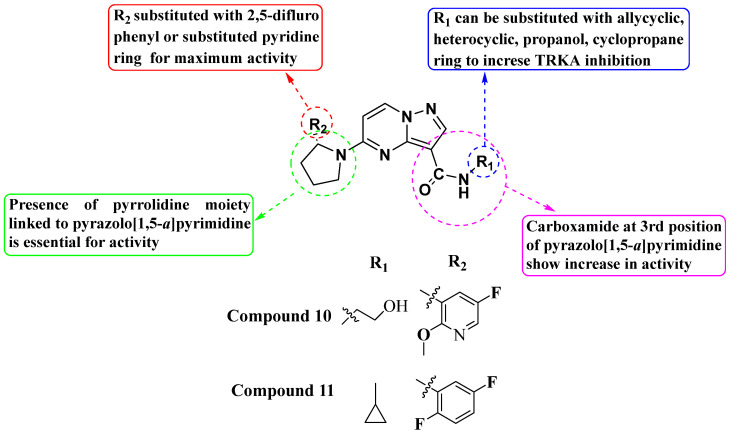
SAR study of carboxamide-linked PP derivatives as TrkA inhibitors.

In 2011, Allen and his team designed and synthesized a new library of novel 3-carboxamide-linked PP derivatives (Figure 6) and evaluated them for their TrkA inhibitory activity. Compounds **10** and **11** emerged as potent TrkA inhibitors, displaying IC_50_ values of 0.2 nM and 0.4 nM, respectively. These compounds also demonstrated inhibitory effects against JAK and TYK in enzymatic assays. The structure–activity relationship (SAR) analysis indicated that the carboxamide moiety at the third position significantly enhances TrkA inhibition. Additionally, substituting alcohol or N-heterocyclic moieties at the NH of the carboxamide further boosts activity. Furthermore, the presence of a 2,5-difluorophenyl or substituted pyridine linked to pyrrolidine at the fifth position of the molecule contributes to increased activity. The study underscores the critical role of the carboxamide moiety and specific substitutions of heterocycles at the -NH group and fifth position in maximizing inhibitory activity against TrkA, while also suggesting potential inhibitory effects against JAK and TYK. Further exploration and substitution of bioactive groups at the -NH of the carboxamide hold promise for developing even more potent Trk inhibitors [79].

**Figure 7:**
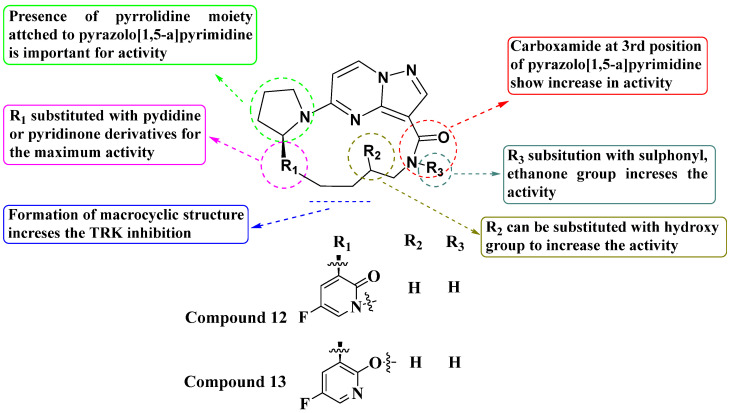
SAR study of macrocyclic PP derivatives as Trk inhibitors.

Andrews and colleagues (2011) synthesized a new series of macrocyclic pyrazolo[1,5-a]pyrimidine derivatives and evaluated their activity using the TrkA Elisa enzyme assay. Within this series, most molecules exhibited potent activity, with IC_50_ values ranging between 1 and 100 nM. Compounds **12** and **13**, which feature a pyridine or pyridinone ring attached to a pyrrolidine moiety, showed particularly strong inhibition. The presence of a carboxamide group significantly enhanced activity, while its absence resulted in reduced activity (IC_50_ values above 100 nM). Substituting the pyrrolidine with other heterocycles such as oxazolidin-2-one decreased activity. The R_3_ group could be substituted with ethenone, sulphonyl, or left unsubstituted to improve activity. In the macrocyclic structure, replacing R_2_ with a hydroxy group maximized activity (see Figure 7). The SAR study concluded that exploring modifications around the carboxamide group could possibly enhance potency and selectivity. Variations in the pyrrolidine moiety and other heterocyclic replacements were suggested to optimize the biological activity [80].

**Figure 8:**
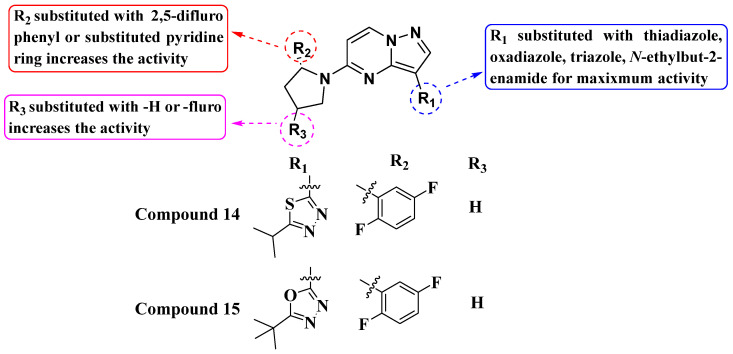
SAR study of heteroaryl-substituted **PP** derivatives as Trk inhibitors.

Kim Moonsoo (2016) reported a new series of heteroaryl-substituted PP derivatives as Trk inhibitors (Figure 8). Compounds **14** and **15** emerged as the most potent in both enzymatic and cell-based assays, with IC_50_ values of less than 10 nM. Kim also evaluated these compounds for cell proliferation in KM12 cell lines, where they demonstrated an IC_50_ value of less than 10 nM. The SAR study revealed that heteroaryl substitution, particularly with thiadiazole, oxadiazole, and triazole at the third position of the PP scaffold, enhances inhibitory activity against Trk receptors. Furthermore, substitution with pyrrolidine at the fifth position further improves inhibition efficacy. The SAR study interprets that the substitution of the pyrrolidine moiety with halogens such as fluorine enhances activity, suggesting specific modifications that could optimize Trk inhibition. It has been anticipated that future efforts may be explored with other halogens or functional groups on the pyrrolidine moiety to further optimize Trk inhibition [81].

**Figure 9:**
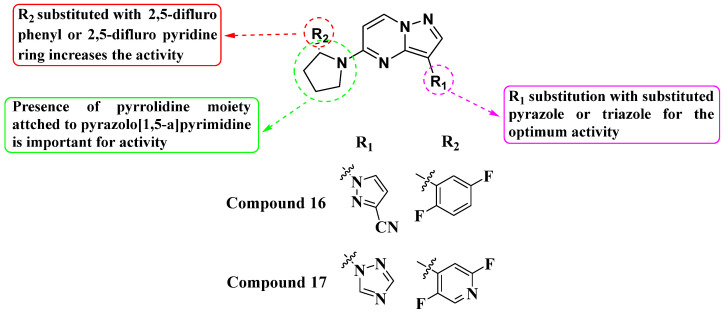
SAR study of pyrazole- and triazole-substituted PP derivatives as TrkA WT inhibitors.

Pal et al. (2019) developed and synthesized a novel library of pyrazole- and triazole-substituted PP derivatives intended as inhibitors of TrkA WT kinase for cancer treatment (Figure 9). They utilized a foundational scaffold similar to the approved drug Larotrectinib, which features a 2,5-difluorophenyl-substituted pyrrolidine linked to the fifth position of pyrazolo[1,5-a]pyrimidine. Various groups were substituted at the third position to optimize activity. Compound **16**, incorporating pyrazole-3-carbonitrile, and compound **17**, incorporating a triazole, exhibited IC_50_ values of >10 nM. The choice of substituents (pyrazole-3-carbonitrile and triazole) at the third position of the scaffold was crucial for achieving high activity against TrkA kinase. This suggests that modifications at this position can significantly influence inhibitory potency. Further exploration and optimization of substituents at the third position and potentially other positions of the scaffold could be pursued to improve potency and selectivity. This iterative process could lead to the discovery of even more effective TrkA inhibitors [82].

**Figure 10:**
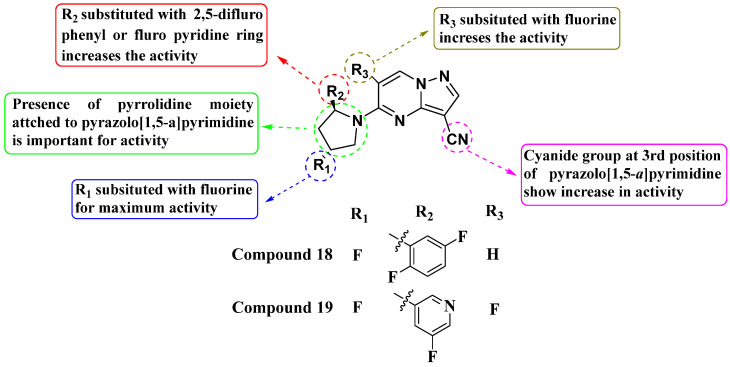
SAR study of nitrile-substituted PP derivatives as NTRK inhibitors.

In 2019, Wenglowsky and colleagues reported on the synthesis of a new library of nitrile-substituted PP compounds designed as NTRK1 inhibitors (Figure 10). Among these, Compounds **18** and **19** emerged as particularly potent, each exhibiting an IC_50_ value of >10 nM against NTRK1 and demonstrating inhibition of KM12 cell proliferation with IC_50_ values of less than 10 nM. Both compounds feature a core pyrrolidine-linked PP scaffold. The introduction of a nitrile substitution at the third position significantly enhanced their activity, while the addition of fluorine to the pyrrolidine ring further improved NTRK1 inhibition. Additionally, substituting fluorine at the sixth position contributed to maximizing their activity. Furthermore, incorporating 2,5-difluorophenyl or pyridine moieties onto the pyrrolidine ring enhanced their efficacy. The SAR findings underscore the importance of structural modifications in optimizing the potency of NTRK1 inhibitors. The combination of nitrile, fluorine, and specific aromatic substitutions demonstrates a synergistic effect in enhancing biological activity. Continued exploration of structural modifications is recommended to fine-tune the potency, selectivity, and pharmacokinetic properties of these compounds. This could involve systematic variation in substituents and scaffold alterations guided by computational modeling and further experimental validation [83].

**Figure 11:**
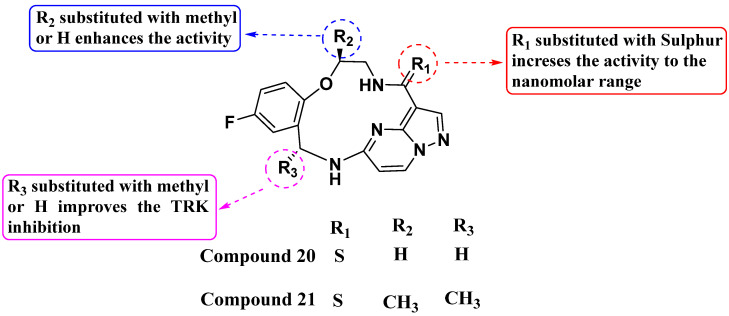
SAR study of macrocyclic PP derivatives as NTRK inhibitors.

Lin et al. (2019) synthesized a novel series of substituted macrocyclic PPs based on the molecule Repotrectinib as NTRK inhibitors (see Figure 11). Compounds **20** and **21** were identified as potent inhibitors of NTRK1, NTRK2, and NTRK3 with IC_50_ values of <0.02 nM each. The macrocyclic structure, incorporating an amide linkage, was crucial for achieving maximum activity. This suggests that the specific spatial arrangement provided by the macrocyclic framework is crucial for binding affinity and biological activity against the NTRK kinases. Substitution of the carbonyl of the amide with sulfur further enhanced NTRK inhibition. This modification likely alters the electronic properties or hydrogen bonding capabilities of the amide linkage, improving the interaction with the target enzymes. Additionally, the introduction of a methyl group on the macrocyclic structures significantly improved their activity, possibly through enhancing hydrophobic interactions [84].

**Figure 12:**
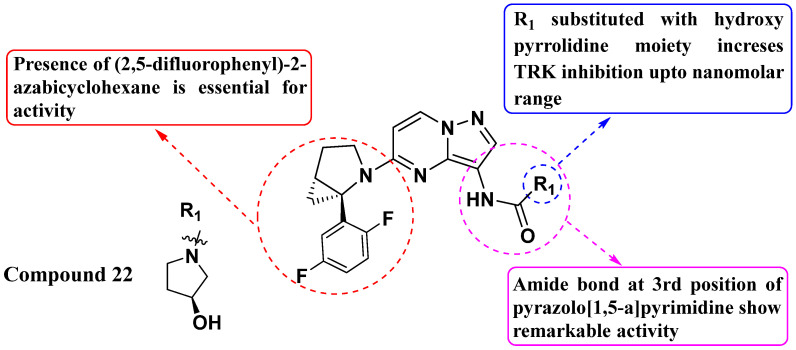
SAR study of 5-azabicyclohexane-substituted PP derivatives as Trk inhibitors.

Hongbin Liu et al. (2019) developed novel derivatives of 5-azabicyclohexane-substituted PP and evaluated their inhibitory activity against Trk fusion mutations. Compound **22** exhibited the highest activity against TrkA, TrkB, and TrkC with IC_50_ values of 3 nM, 14 nM, and 1 nM, respectively (see Figure 12). Furthermore, compound **22** demonstrated potent inhibition of KM12 cell proliferation with an IC_50_ of 1 nM. Importantly, the presence of a 2,5-difluorophenyl substitution at the fifth position of the azabicyclohexane scaffold was identified as crucial for optimal inhibitory activity against Trk receptors. Additionally, the presence of a hydroxypyrrolidine moiety further enhanced the activity. This SAR information is crucial for guiding future compound optimization efforts. Continued optimization of the compounds based on the SAR findings could lead to the development of even more potent inhibitors with improved pharmacokinetic properties and reduced off-target effects [85].

**Figure 13:**
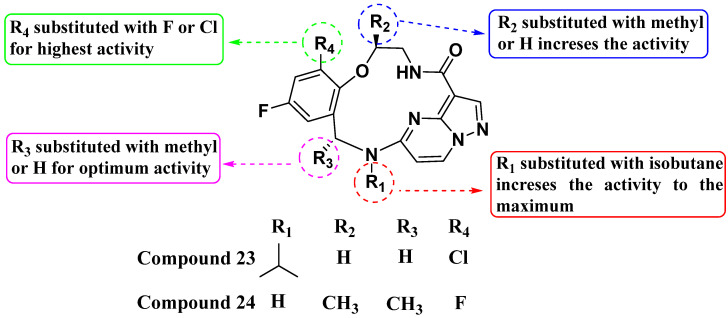
SAR study of macrocyclic kinase inhibitors based on the PP scaffold.

Cui et al. (2020) introduced a novel series of substituted macrocyclic kinase inhibitors based on the PP scaffold (Figure 13). Compounds **23** and **24**, inspired by Repotrectinib, feature a macrocyclic structure with amide substitutions and demonstrate inhibition of cell proliferation in TRKA KM12 cells with IC_50_ values of 0.1 and 0.2 nM, respectively. These compounds also exhibit activity against the JAK2 SET2 cell line [**23** = 1479 nM; **24** = 3.9 nM] and the BTK cell line [**23** = 179 nM; **24** = 14.25 nM]. The introduction of a macrocyclic structure based on the PP scaffold was crucial for enhancing kinase inhibition. Amide substitutions significantly enhance activity against TRKA, indicating the importance of specific interactions conferred by the amide bonds. The attachment of a methyl group to the macrocycle further enhances inhibition potency, suggesting that structural modifications can fine-tune and improve biological activity [86].

**Figure 14:**
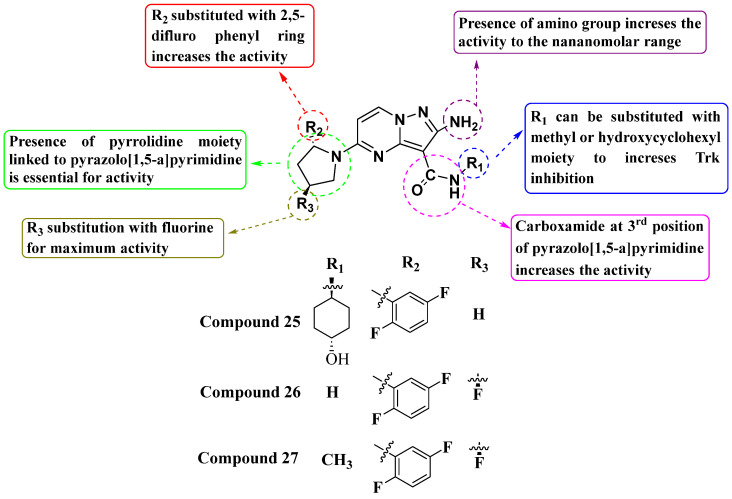
SAR study of amino PP derivatives as Trk inhibitors.

Li Zhu et al. (2020) synthesized and evaluated a novel series of substituted amino PP compounds as inhibitors of the neurotrophic factor Trk. They tested a total of 52 molecules for their ability to inhibit Trk activity, identifying compounds **25**, **26**, and **27** as the most potent with IC_50_ values of less than 1 nM (see Figure 14). These compounds effectively inhibit TrkA, TrkB, and TrkC with an IC_50_ of 1 nM. The structure–activity relationship (SAR) analysis indicates that substituting the pyrrolidine moiety with 2,5-difluorophenyl and fluorine enhances activity significantly. Additionally, the presence of an amino group at the second position and a carboxamide at the third position further enhance Trk inhibition. Subsequent observations suggest that substituting the -NH group of the carboxamide with a methyl or hydroxy cyclohexyl moiety contributes to maximizing activity. While the compounds showed potent inhibitory activity, future studies should focus on the exploration of modification on the other substituents in the place of methyl or hydroxy cyclohexyl for better inhibition [87].

**Figure 15:**
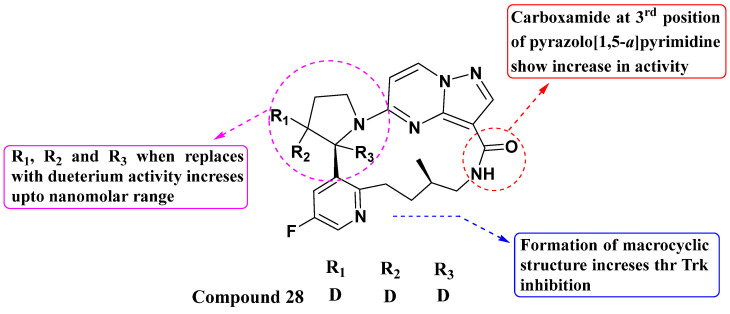
SAR study of substituted PP macrocyclic derivatives as Trk inhibitors.

Yihan Wang et al. synthesized and evaluated a series of novel substituted PP macrocyclic compounds against all three Trks in 2020 (Figure 15). Among these, compound **28** demonstrated the highest activity with IC_50_ values of 0.17 nM, 0.07 nM, and 0.07 nM against TrkA, TrkB, and TrkC, respectively. The macrocyclic structure offers advantages over the first drug Larotrectinib. Macrocycles often have improved binding affinity and selectivity due to their conformational rigidity and ability to form specific interactions with the target receptor. The presence of a carboxamide at the third position enhances Trk inhibitory activity. This suggests that specific modifications at this position could be further explored to optimize potency and selectivity. Additionally, replacing the hydrogen atom of the pyrrolidine with a deuterium moiety helps achieve activity in the nanomolar range. This isotopic substitution technique can potentially improve the pharmacokinetic properties of the compound, such as enhancing metabolic stability or altering pharmacokinetic profiles [88].

**Figure 16:**
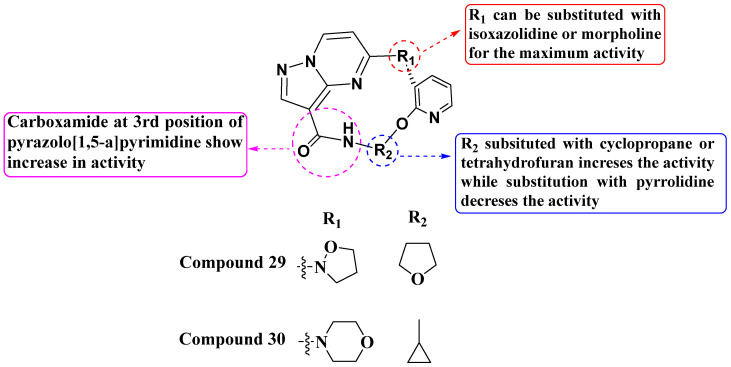
SAR study of macrocyclic PP derivatives as selective Trk inhibitors.

Wang and his colleagues, in 2021 and 2022, discovered a novel series of macrocyclic PP derivatives as selective Trk inhibitors. Among them, compounds **29** and **30** exhibited the highest inhibitory activity against TrkA, TrkC, ALK, and ROS (Figure 16). Compound **29** (TRK A = 0.6 nM, TRK C = 0.1 nM, ALK = 901 nM, Ros1 = 2.2 nM) and compound **30** (TRK A = 1.61 nM, TRK C = 0.05 nM, ALK = 10.40 nM, Ros1 = 0.16 nM) showed promising potency as TrkA, TrkC, ALK, and ROS inhibitors. Wang also conducted cell proliferation assays using the Ba/F3 LMNA-NTRK1-WT stably transfected cell line. The SAR (Structure–Activity Relationship) study revealed that the presence of a carboxamide group at the third position enhances Trk inhibition. Additionally, Wang and his team synthesized and evaluated substituted pyridine-linked isoxazolidine or morpholine derivatives in place of pyrrolidine within the macrocyclic PP derivatives. This modification led to achieving inhibition in the nanomolar range. Furthermore, continued exploration of structural modifications, including cyclic groups such as cyclopropane and tetrahydrofuran, could lead to compounds with improved pharmacokinetic properties and selectivity profiles [89,90].

**Figure 17:**
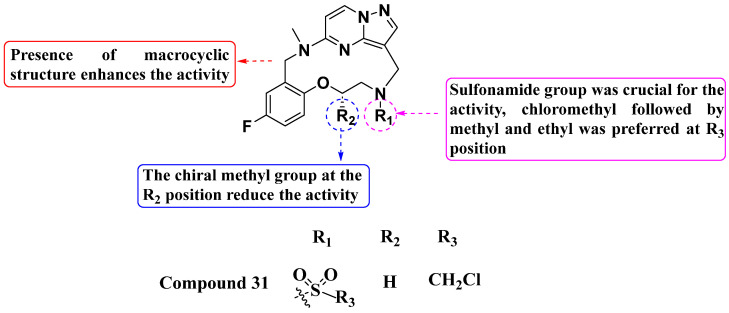
SAR study of sulfonamide-substituted macrocyclic PP derivatives as Trk inhibitors.

Zhang, Zhou, and their colleagues (2021) reported a novel macrocyclic analog as a potent TRK inhibitor (Figure 17). Compound **31** displayed superior TRKG595R kinase inhibition with an IC50 value of 13.1 nM and moderate antiproliferative activity against the Ba/F3-LMNA-NTRK1 cell line with an IC_50_ value of 0.080 µM. Furthermore, it exhibited better kinase inhibition (IC_50_ value of 0.646 µM) compared to the standard LOXO-101 in the Ba/F3-LMNA-NTRK1-G595R cell line. SAR studies revealed that substitution with a sulfonamide group at the R_1_ position is crucial for TRKG595R inhibition. Conversely, a sulfonamide with an amide linker at R_1_ was not tolerated and led to decreased activity. At the R_2_ position, the chiral methyl group did not confer activity, and substitution with urea also did not exhibit TRKG595R inhibition. Increasing the carbon chain length tended to sustain strong inhibition of TRKA; however, inhibition against the TRKG595R variant was comparatively less effective than with other compounds. Compounds with a two-carbon side chain showed significantly higher activity compared to those with a three-carbon side chain. At the R_3_ position, chloromethyl substituents were preferred, followed by methyl and ethyl groups. Further exploration of R_1_ substitutions, particularly variations of the sulfonamide group, could enhance potency and selectivity against TRKG595R while maintaining overall kinase inhibition [91].

**Figure 18:**
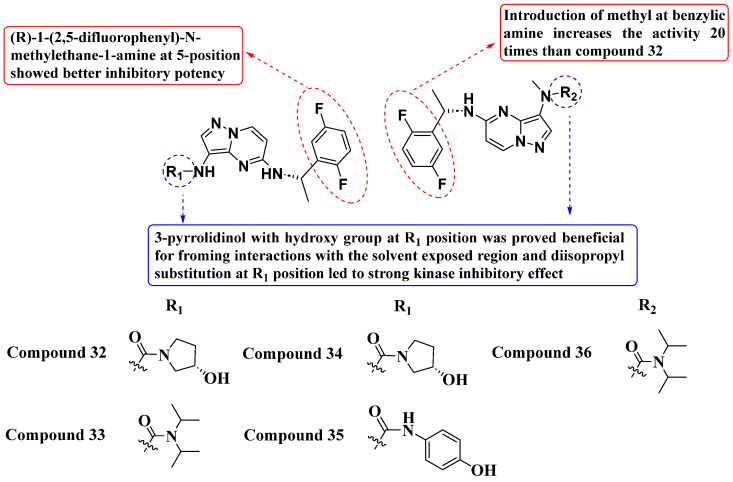
SAR study of novel PP-based TrkA, TrkB, and TrkC inhibitors.

Tang and Fan et al. (2021) developed a novel series of PP-based TRK inhibitors (Figure 18). Among these, compound **32** exhibited IC_50_ values of 1.9 nM, 3.1 nM, and 2.3 nM against TrkA, TrkB, and TrkC, respectively. Compound **33** showed IC_50_ values of 3.2 nM, 5.5 nM, and 3.3 nM, compound **34** showed IC_50_ values of 1.8 nM, 4.1 nM, and 2.3 nM, compound 35 showed IC50 values of 2.5 nM, 3.1 nM, and 2.6 nM, and compound **36** showed IC_50_ values of 1.4 nM, 2.4 nM, and 1.9 nM. These values indicate significant inhibition against TrkA, TrkB, and TrkC compared to the standard drug Larotrectinib, which showed IC_50_ values of 1.2 nM, 2.1 nM, and 2.1 nM, respectively. Extensively, The SAR studies highlighted the importance of a 3-pyrrolidinol scaffold with a hydroxy group at the R1 position for effective interaction with the solvent-accessible domain of Trk receptors. Substitution at R1 and modifications at the 5-position (introduction of (R)-1-(2,5-difluorophenyl)-N-methylethane-1-amine) significantly enhanced inhibitory potency. Additionally, methyl substitution at the benzylic amine notably increased inhibitory activity by 20 times compared to compound 33, demonstrating the sensitivity of the compounds to small structural modifications [92].

**Figure 19:**
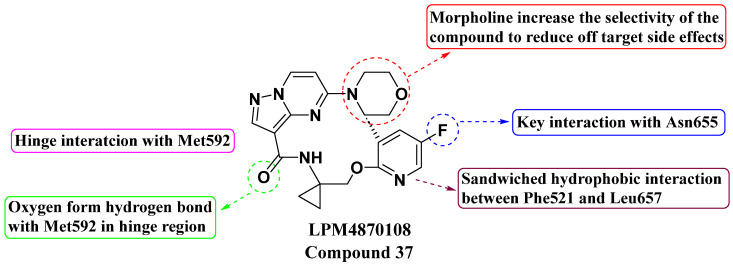
SAR study of second-generation macrocyclic PP derivatives as Pan-Trk inhibitors.

Wang, Tian, et al. (2021) discovered a second generation of macrocyclic compounds as potent Pan-Trk kinase inhibitors through structural modification and optimization, resulting in enhanced physicochemical and oral pharmacokinetic parameters (see Figure 19). Compound **37**, known as LPM4870108, exhibited significant activity against both wild-type and mutant forms of TrkA and TrkC. In vitro evaluation of compound **37** demonstrated inhibition in enzymatic assays against TrkA, TrkAG595R, TrkAG667C, TrkC, ALK, and Ros1 with IC_50_ values of 2.4 ± 0.3 nM, 3.5 ± 0.5 nM, 2.3 ± 0.3 nM, 0.2 ± 0.1 nM, 182.0 ± 14.2 nM, and 1.0 ± 0.3 nM, respectively. Cellular assays against Ba/F3 TTRK, Ba/F3 TrkAG595R, Ba/F3 TrkAF589L, Ba/F3 TrkAG667A, Ba/F3 TrkCG623R, and Ba/F3 SLC34A2-Ros1 showed IC_50_ values of 0.6 ± 0.1 nM, 7.7 ± 0.5 nM, 4.9 ± 0.2 nM, 0.8 ± 0.1 nM, 2.7 ± 0.2 nM, and 2.3 ± 0.3 nM, respectively. LOXO-195 and TPX-0005 were used as standards. The SAR study identified key structural features crucial for potency and selectivity. The pyrazolo[1,5-*a*]pyrimidine moiety was essential for forming a hinge interaction with the Met592 residue, influencing binding affinity. The addition of a morpholine group at a specific position improved selectivity by reducing off-target effects. Fluorine incorporation enhanced interactions with Asn655, while a pyridine ring supported hydrophobic interactions, contributing to overall potency. The lipophilicity of compound 37 was increased, leading to enhanced metabolic stability and a favorable in vivo profile with reduced toxicity [93].

**Figure 20:**
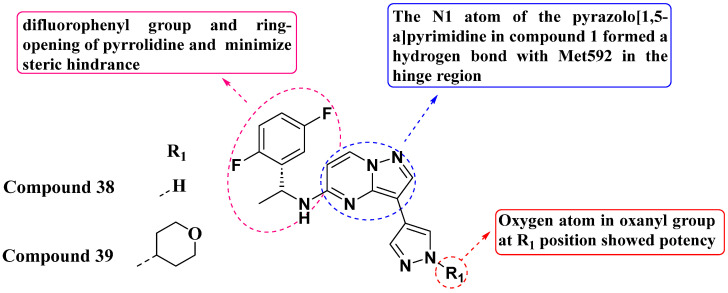
SAR study of 3-Pyrazolyl-substituted PP derivatives as Trk inhibitors.

In addressing acquired resistance in second-generation inhibitors, Huang et al. (2022) employed ring-opening scaffold-hopping strategies to design and synthesize a series of PP analogs substituted with 3-Pyrazolyl, replacing the 3-pyrrolidinol moiety (Figure 20). Pyrazole was utilized to optimize molecular orientation and minimize steric conflicts. Among these compounds, compound **38** exhibited potent kinase inhibitory activity against TrkAF589L and TrkAG595R mutations. However, it did not enhance enzymatic inhibition against TrkAG667C. Structural refinement of compound **38** resulted in the development of compound **39**, which emerged as the most potent within the series. Compound 39 demonstrated IC_50_ values of 2.3 nM, 0.4 nM, and 0.5 nM against the TrkAF589L, TrkAG667C, and TrkAG595R cell lines, respectively, surpassing Selitrectinib as the standard drug. Structure–activity relationship (SAR) studies revealed that the presence of oxygen in the oxanyl group contributed to potency, occupying the mutation site of x-DFG. Additionally, both the difluoro phenyl group and the ring-opening of the pyrrolidine ring optimized molecular orientation to minimize steric hindrance. The N1 atom of pyrazolo[1,5-*a*]pyrimidine formed a hydrogen bond with the amino acid Met592 in the hinge region. This insight into SAR underscores the importance of molecular interactions in designing potent kinase inhibitors. Compound **39** exhibited improved pharmacokinetic properties and demonstrated significant antitumor activity, achieving tumor growth inhibition rates of 97% at 30 mg/kg/dose and 73% at 100 mg/kg/dose in xenograft mouse models for both TrkAWT and TrkAG667C mutations [94].

**Figure 21:**
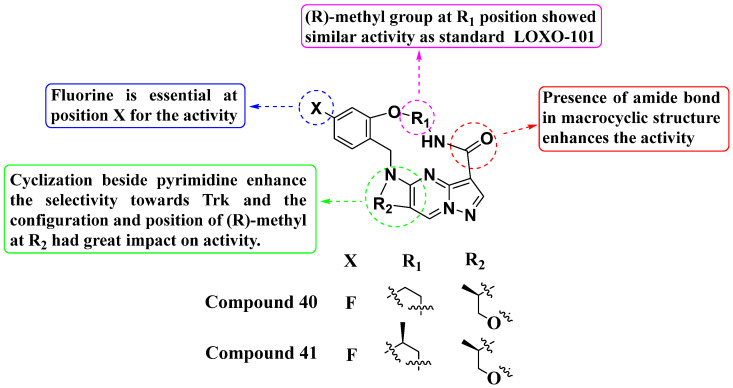
SAR study of second-generation PP derivatives as Trk inhibitors.

Second-generation inhibitors such as TPX-0005 (Repotrectinib, Augtyro, Bristol Myers Squibb Company, Princeton, New Jersey, US) have proven more effective than first-generation inhibitors. First-generation Trk inhibitors encountered issues with drug resistance mutations. In 2022, Fan et al. designed and synthesized a new series of second-generation PP analogs, which were demonstrated to be effective as Trk inhibitors (Figure 21). Compounds **40** (with IC_50_ values of 1.40 nM and 1.80 nM) and **41** (with IC_50_ values of 0.86 nM and 6.92 nM) showed enhanced inhibition of TrkA and Trk-AG595R, respectively. TPX-0005 was used as a positive control. Compound **41** also displayed inhibitory activity against ALK with an IC_50_ value of 350 nM, yet selectively inhibited Trk, potentially reducing toxic effects. Structure–activity relationship (SAR) studies revealed that a fluorine atom at position X is crucial for activity. Shifting the fluorine position on the benzene ring and cyclization adjacent to the pyrimidine moiety did not significantly affect TPX-0005’s activity but enhanced its selectivity. The configuration and position of the methyl group at R_2_ notably influenced its activity. A (R)-methyl group at the R_1_ position exhibited activity comparable to the standard LOXO-101. Overall, these findings underscore the promising potential of these second-generation Trk inhibitors, offering improved efficacy and selectivity over their predecessors [95].

**Figure 22:**
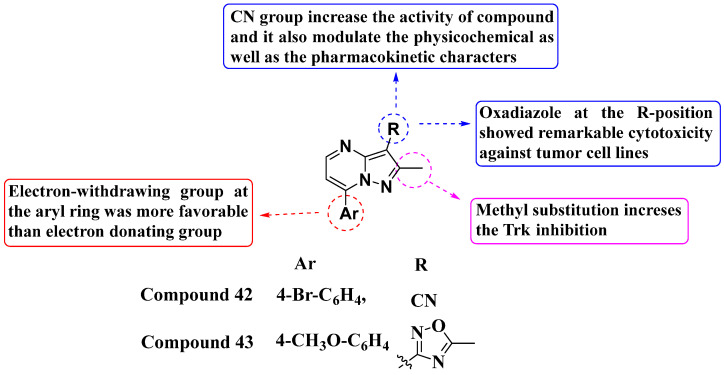
SAR study of 7-aryl-3-substituted PP derivatives as RTK and STK inhibitors.

In 2023, Al-Qadhi and his colleagues designed and synthesized a novel series of 7-aryl-3-substituted PP analogs, evaluating their inhibition against RTK and STK (Figure 22). Compound **42** demonstrated potent enzymatic inhibition against TrkA and ALK2, with IC_50_ values of 0.087 μM and 0.105 μM, respectively. This compound also exhibited robust antiproliferative activity against two cell lines, KM12 (IC_50_ = 0.82 μM) and EKVX (IC50 = 4.13 μM). Compound **43** also showed significant potency against TrkA in the sub-micromolar range. Additionally, it demonstrated antiproliferative activity against various cell lines: MCF7 (IC_50_ = 3.36 μM), HCT116 (IC_50_ = 1.40 μM), and EKVX (IC_50_ = 3.49 μM), with notable safety profiles. Larotrectinib was used as a standard reference. The electron-withdrawing group at the seventh position of the aryl ring was found to be more favorable than the electron-donating group. The cyano (CN) group at the third position played a crucial role in enhancing the activity of the compounds. Moreover, the oxadiazole substitution at the R-position showed remarkable cytotoxicity against various tumor cells [96].

**Figure 23:**
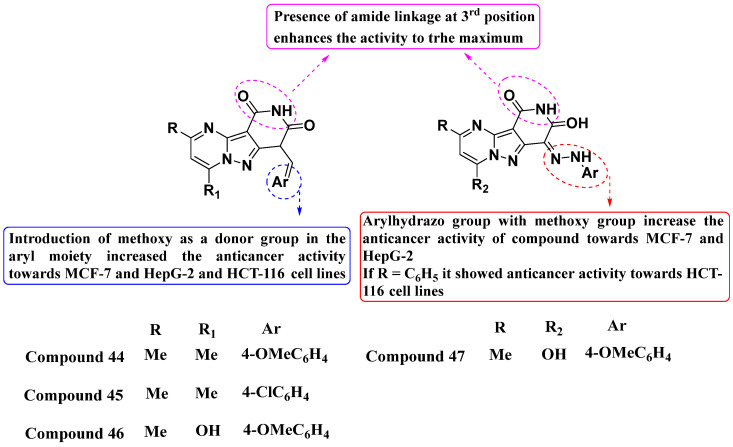
SAR study of fused PP derivatives as TrkA inhibitors.

Recently, Metwally et al. (2024) developed a new series of molecules and evaluated their efficacy against MCF7, HepG2, and HCT116 cell lines (see Figure 23). Compounds **44** and **47** exhibited the most promising inhibitory activity against TrkA, with IC_50_ values of 0.064 ± 0.0037 μg/mL and 0.047 ± 0.0027 μg/mL, respectively, while Larotrectinib served as the positive control. Notably, all potent compounds (**44**, **45**, **46**, and **47**) induced cell cycle arrest at the G2/M phase. Compound **46** demonstrated the highest apoptosis rate (36.72%) among them, surpassing compounds **44** (34.70%), **45** (21.14%), and **47** (26.54%). Structure–activity relationship (SAR) studies revealed that the introduction of a donor group, such as a methoxy group in the aryl moiety, enhanced the anticancer activity across the MCF-7, HepG-2, and HCT-116 cell lines. Furthermore, the incorporation of an Arylhydrazo group with the methoxy group notably increased anticancer efficacy, specifically against MCF-7 and HepG-2 cells. Interestingly, compounds where R = C_6_H_5_ demonstrated significant anticancer activity against the HCT116 cell line. Based on SAR findings, further optimizing the chemical structure could improve potency, selectivity, and pharmacokinetic properties. Exploration of additional substituents or modifications could enhance efficacy against specific cancer types [97].

## 4. Summary

In this review, the development of pyrazolo[1,5-a]pyrimidine derivatives as Trk inhibitors was discussed in detail. Following the identification of NTRK1 as an oncogene in 1982 by Mariano Barbacid and colleagues, the actual development of NTRK inhibitors began in 2015. Currently, only three drugs are marketed as Trk inhibitors, two of which contain a pp nucleus. The potency of the pp nucleus in Trk inhibition has drawn the attention of researchers, leading to the development of many small molecules based on the pp core nucleus as Trk inhibitors. Several macrocyclic molecules containing the pp core nucleus are undergoing clinical trials as Trk inhibitors. This review provides insights into the SAR studies of several PP derivatives, highlighting that substitution at the third and fifth positions of PP was essential for Trk inhibition. Compounds with an amide or carboxamide bond at the third position tend to exhibit Trk inhibition in the nanomolar range. Substituting a pyrrolidine ring at the fifth position offers advantages over other rings. The pyrrolidine ring, especially when electronegatively substituted, enhances Trk inhibition. Some compounds with substitutions at positions other than the third and fifth also demonstrate excellent inhibitory effects. The important structural features alongside potency values are summarized in Table 1.

## 5. Conclusions

This article discusses the significance of pyrazolo[1,5-a]pyrimidine derivatives as Trk inhibitors, which are crucial for treating solid tumors. Extensive research into the pyrazolo[1,5-a]pyrimidine nucleus indicates significant potential for diverse opportunities in anticancer drug discovery. Trk inhibitors with this core structure, such as Larotrectinib sulfate, Entrectinib, Repotrectinib, and Selitrectinib, are already on the market. However, the effectiveness of some of these is limited due to resistance mutations. A macrocycle-based analog and several other TRK inhibitors are currently undergoing clinical trials. This study concludes that structural modifications of the pyrazolo[1,5-a]pyrimidine nucleus have led to compounds **20** and **21**, which exhibit NTRK IC_50_ values >0.02 nM. Compounds **23** and **24** show TRKA (KM12 cell) inhibition with IC_50_ values of 0.1 nM and 0.2 nM, respectively. These compounds could be further optimized to develop potent leads. This article provides comprehensive synthetic and SAR-based information on the pyrazolo[1,5-a]pyrimidine core to aid in the development of potent and novel derivatives as TRK inhibitors in medicinal chemistry. Therefore, further exploration of the pyrazolo[1,5-a]pyrimidine scaffold is essential to identify additional TRK inhibitors with enhanced clinical applications in the future.

## Figures and Tables

**Table 1:** The chemical structure features and IC_50_ value of potent compounds.

Compd. No.	Chemical Structure	Chemical Structure Feature	IC_50_ Value
**8**	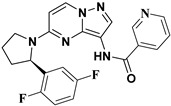	The presence of a pp core with a 2,5-difluorophenyl-substituted pyrrolidine at the fifth position, along with an amide bond at the third position.	1.7 nM
**10**	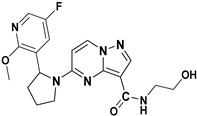	The presence of a pp core with a 2,5-difluoropyridine-substituted pyrrolidine at the fifth position, along with a hydroxyethyl acetamide at the third position.	0.2 nM
**12**	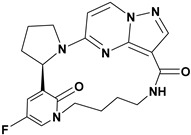	The presence of a pp core with substituted pyrrolidine at the fifth position, along with a carboxamide at the third position forming a macrocyclic structure.	>100 nM
**14**	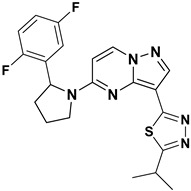	The presence of a pp core with a 2,5-difluorophenyl-substituted pyrrolidine at the fifth position, along with a thiadiazole bond at the third position.	>10 nM
**18**	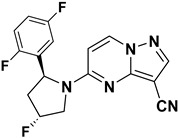	The presence of a pp core with a 2,5-difluorophenyl-substituted pyrrolidine at the fifth position, along with cyanide at the third position.	>10 nM
**20**	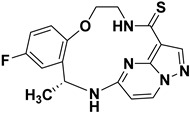	The presence of a macrocyclic pp core with fluorophenyl attached to amine substituted at the fifth position, along with a carbothioamide at the third position.	>0.02 nM
**32**	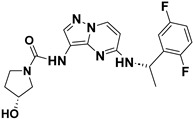	The presence of a pp core with amine substituted at the fifth position, along with a pyrrolidine carboxamide at the third position.	1.9 nM, 3.1 nM, 2.3 nM

## Data Availability

Not applicable

## Abbreviations

PP	Pyrazolo[1,5-*a*]pyrimidine
TRK	Tropomyosin Receptor Kinases
SAR	Structure-Activity Relationship
FDA	Food and Drug Administration
NGF	Nerve Growth Factor
BDNF	Brain-Derived Neurotrophic Factor
NT3	Neurotrophin-3
AML	Acute Myeloid Leukemia
CNS	Central Nervous System
CDK	Cyclin-Dependent Kinases
RTK	receptor tyrosine kinases
EGFRs	Epidermal Growth Factor Receptors
FGFRs	Fibroblast Growth Factor Receptors
ALK	Anaplastic lymphoma kinase
ROS1	ROS proto-oncogene 1, receptor tyrosine kinase
